# Supplementary material for: Neuroprotective Action of Tacrolimus before and after Onset of Neonatal Hypoxic–Ischaemic Brain Injury in Rats
Source: Cells. 2023 Nov 20;12(22):2659. doi: 10.3390/cells12222659 (PMC10669941; doi:10.3390/cells12222659)
Supplement: Supplementary file 1 [file cells-12-02659-s001.zip › cells-2681989-supplementary.pdf]

Supplementary File S1:

Table S1. Experimental groups to assess the immunosuppressive effect of tacrolimus administered for three days until post mortem on PND 11.

Experimental group	Number of pups	Pup sex (M/F)
**PBS**	14	7/7
**0.025 mg/kg/day tacrolimus**	10	4/6
**0.05 mg/kg/day tacrolimus**	9	4/5
**0.25 mg/kg/day tacrolimus**	8	4/4
**0.5 mg/kg/day tacrolimus**	4	2/2
**1.0 mg/kg/day tacrolimus**	5	3/2

Table S2. Experimental groups to assess the long-term safety of tacrolimus administered daily from PND 7 to PND 50.

Experimental group	Number of pups	Pup sex (M/F)
**PBS**	16	8/8
**0.025 mg/kg/day tacrolimus**	8	4/4
**0.05 mg/kg/day tacrolimus**	9	5/4
**0.1 mg/kg/day tacrolimus**	7	3/4
**0.25 mg/kg/day tacrolimus**	6	3/3
**0.5 mg/kg/day tacrolimus**	6	3/3
**1.0 mg/kg/day tacrolimus**	7	4/3

Table S3. Experimental groups to assess the neuroprotective action of tacrolimus.

<table>
<tr><th>Experimental group</th><th>Number of pups (M/F)
Pre-HI tacrolimus or PBS started 3 days before HI</th><th>Number of pups (M/F)
Post-HI tacrolimus or PBS started 12 hours after HI</th></tr>
<tr><td>Sham + PBS</td><td colspan="2">11 (6/5)</td></tr>
<tr><td>HI + PBS</td><td colspan="2">10 (6/4)</td></tr>
<tr><td>HI + 0.025 mg/kg/day tacrolimus</td><td>8 (4/4)</td><td>8 (4/4)</td></tr>
<tr><td>HI + 0.05 mg/kg/day tacrolimus</td><td>8 (4/4)</td><td>8 (4/4)</td></tr>
<tr><td>HI + 0.1 mg/kg/day tacrolimus</td><td>7 (5/2)</td><td>8 (4/4)</td></tr>
<tr><td>HI + 0.25 mg/kg/day tacrolimus</td><td>8 (3/6)</td><td>9 (5/4)</td></tr>
<tr><td></td><td></td><td>7 (3/4)</td></tr>
<tr><td></td><td></td><td>6 (3/3)</td></tr>
</table>

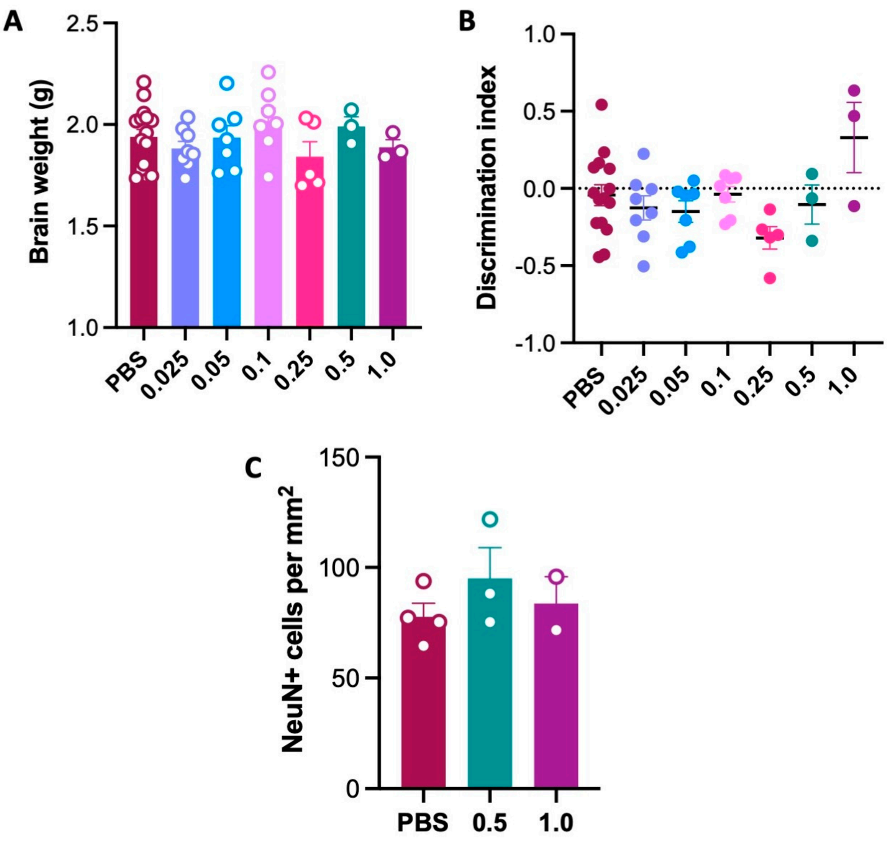

Figure S1. Tacrolimus has no long-term detrimental effect on the brain or cognitive function. (A) Brain weight at PND50 following long term daily treatment with tacrolimus, n = 3–16 (B) Novel object recognition test performed at PND50 shows no cognitive deficits in animals that received daily tacrolimus, n = 3–15. (C) Neuron cell count in the CA3 region of the hippocampus was not reduced with higher doses of tacrolimus when a small subset of animals were compared to PBS controls, n = 2–4.
